# The Association Between Female Breast Size, Backache, and Quality of Life in Young Women: A Cross-Sectional Study

**DOI:** 10.3390/medicina61081353

**Published:** 2025-07-26

**Authors:** Raphael Lotan, Natali Marmor, Sharon Weiss, Mojahed Sakhnini, Oded Hershkovich

**Affiliations:** 1 Department of Orthopedic Surgery, Wolfson Medical Center, Holon 5822012, Israelmojahed.sakh@gmail.com (M.S.); 2 Gray Faculty of Medical and Health Sciences, Tel Aviv University, Tel Aviv 6997801, Israel; natalima@wmc.gov.il (N.M.); dareznee@walla.co.il (S.W.); 3 Department of Plastic Surgery, Wolfson Medical Center, Holon 5822012, Israel

**Keywords:** female, breast, back pain, size, mammaplasty

## Abstract

*Background and Objectives*: The association between female breast size and spinal back pain is widely suggested in clinical practice but remains insufficiently quantified in general, non-surgical populations in the scientific literature. Larger breasts may increase biomechanical strain on the spine, contributing to musculoskeletal pain and reduced quality of life. This study aimed to evaluate the association between breast size and back pain in a general orthopedic population of young women. *Materials and Methods*: A cross-sectional study was conducted among 200 women aged 18–36 who attended orthopedic clinics for non-spinal complaints. Data were collected via structured telephone questionnaires, including demographics, self-reported breast size (cup and band), pain characteristics, and SF-12 quality of life scores. Binary logistic regression, ANOVA, and chi-square analyses assessed associations between breast size, pain presence, severity, and functional outcomes. *Results*: Back pain prevalence increased with breast size: only 4.9% of B cup participants reported backache, compared to 85% of DD/E cup participants. VAS scores rose from 0.3 ± 1.6 (B cup) to 6.0 ± 2.9 (DD/E cup). Each 1 cm increase in band length raised the odds of back pain by 19.8% (OR = 1.198, *p* < 0.001), while large cup size was associated with up to 12-fold increased odds of pain. Larger breast size was also significantly associated with work limitations and social impairment. *Conclusions*: Breast size was strongly associated with the presence and severity of back pain, particularly in the thoracic and cervical regions. Clinicians should consider breast size in the assessment of backache, and reduction mammaplasty may have therapeutic value beyond aesthetics.

## 1. Introduction

Back pain is one of the most common musculoskeletal complaints among women, with the majority experiencing at least one episode of significant backache during their lifetime [1]. Among the various risk factors, the role of breast size, particularly macromastia (excessively large breasts), has been the subject of longstanding debate in both orthopedic and plastic surgery literature. Other causes of back pain include gynecological origins, such as endometriosis, salpingitis, or uterine fibroids; urologic origins, such as urinary tract diseases, digestive tract diseases, and retropleural and retroperitoneal diseases; and even malignancies [2].

Multiple cross-sectional studies have reported a significant association between large breast size and increased prevalence and intensity of back pain. Women with hypertrophic breasts (breast volume > 1200 mL) consistently report higher levels of musculoskeletal pain across the upper torso, including the upper and lower back, shoulders, and neck [3]. Larger breast size has been shown to increase thoracic flexion torque, potentially leading to greater mechanical strain and postural imbalance [3,4]. These postural changes have also been documented, including increased thoracic kyphosis and decreased scapular and shoulder function [4,5,6].

The orthopedic literature similarly reflects these concerns. Schinkel-Ivy and Drake found that larger breast size influences spinal kinematics and activates postural musculature differently, which may contribute to chronic discomfort [7]. Hershkovich et al., in a study of over 800,000 adolescents, demonstrated a significant association between body mass index (BMI), height, and the prevalence of back pain, further supporting the role of body morphology in musculoskeletal symptoms [8]. Spencer et al. later showed a statistically significant correlation between upper back pain and breast size in postmenopausal women [9].

Nevertheless, the evidence is not unequivocal. While several studies confirm mechanical and postural links between larger breast size and back pain [4,7,10], others highlight that breast size alone may not fully account for musculoskeletal sensitivity and pain perception [11,12]. For instance, Spencer et al. (2022) found no direct association between breast size and upper back tissue sensitivity, indicating multifactorial origins of pain [11]. Similarly, Petronilla et al. (2024) observed increased kyphosis with larger breasts but no direct correlation with thoracic pain [5].

Reduction mammaplasty has emerged as an effective intervention for alleviating back pain in patients with macromastia. Multiple studies have demonstrated improvements in pain, posture, and physical function following surgery [6,13,14,15]. Foreman et al. observed subjective reductions in pain and biomechanical load post-surgery despite no statistically significant changes in objective mechanical measures [12]. A systematic review and meta-analysis by Mian et al. (2020) confirmed the trend of improvement across several studies, though it also emphasized the limited quality and quantity of available evidence [14].

In summary, the literature strongly suggests a relationship between larger breast size and increased back pain, driven by biomechanical, postural, and functional mechanisms. However, limitations in study design, population characteristics, and variable control have left the subject open to further investigation. Notably, most existing data derive from surgical cohorts or patients actively seeking breast reduction, introducing considerable potential bias.

The present study aims to evaluate the association between self-reported female breast size and the presence of back pain in a population of young, otherwise healthy women who have not sought breast reduction surgery and have not been referred to specialized spine clinics. This approach aims to reduce selection bias and provide clearer insight into whether breast size independently correlates with back pain prevalence and severity.

## 2. Materials and Methods

This cross-sectional telephone-based study was conducted among 200 young female patients who attended hospital orthopedic clinics (excluding spine clinics) between 2015 and 2020 for reasons unrelated to back pain. Data were collected via structured telephone questionnaires, SF-12, following verbal informed consent. Women who had visited orthopedic subspecialty clinics (hand, foot, trauma, sports medicine) were included.

The sample size for this cross-sectional study was determined based on expected differences in the prevalence of back pain between women with smaller and larger breast sizes, as reported in the existing literature. Assuming a conservative prevalence difference of 10% versus 50%, with a desired power of 80% and a two-sided significance level of 5%, a minimum of approximately 35 participants per group would be required to detect a statistically significant difference using standard sample size formulas for comparing proportions. Given that breast size was assessed across multiple categories (B, C, D, DD/E, DDD/F), and considering the need for subgroup analyses and multivariable logistic regression to control for potential confounders such as age and weight, a total sample size of 200 participants was selected.

The exclusion criteria consisted of a history of spinal trauma, vertebral fractures or spinal tumors, and congenital or developmental spinal deformities (e.g., scoliosis, kyphosis). Prior spinal or breast augmentation surgery was an exclusion criterion. Diagnoses of gynecological, urological, or gastrointestinal pathology were excluded. Pregnancy during the study or within one year before the survey was an exclusion criterion.

The measured parameters included breast size, self-reported using standard bra measurements (cup size and band size), age, and BMI based on self-reported height and weight. Back pain characterization had several categories: Subjective: pain without supportive imaging findings; Pain Location: thoracic spine, lumbar spine, or both; Pain Intensity: assessed via visual analog scale (VAS); Frequency: number of pain episodes per year. The impact on quality of life was evaluated using the SF-12 questionnaire.

Data were extracted from the hospital’s computerized records, including outpatient charts and archived files. Participant identities were anonymized using serial codes.

This study was reviewed and approved by the Institutional Review Board (IRB) of the Medical Center. All procedures involving human participants were conducted following the ethical standards of the institutional and national research committees and the 1964 Helsinki Declaration and its later amendments. Verbal informed consent was obtained from all participants before inclusion in the study, and all data were anonymized to protect patient confidentiality.

Statistical Analysis: Descriptive statistics were used to summarize demographic, clinical, and quality of life variables across breast size categories. Continuous variables were reported as means and standard deviations, and comparisons between groups were performed using the Student’s *t*-test or one-way ANOVA where appropriate. Categorical variables were analyzed using the chi-square test to assess associations between cup size and the presence of pain or functional impairments. Binary logistic regression was used to evaluate the relationship between back pain (overall and by spinal region) and continuous predictors, including age, weight, height, and bra band length, as well as categorical predictors, such as cup size. Multivariate logistic regression models were constructed to identify independent predictors of back pain while controlling for potential confounders. Subgroup analyses were conducted among participants reporting back pain to examine the association between breast size and SF-12 functional domains. A *p*-value < 0.05 was considered statistically significant in all tests. Statistical analyses were conducted using standard software.

## 3. Results

A total of 200 female participants aged 18 to 36 years were included in the analysis. Seventy-nine female participants reported back pain. The participants were categorized by self-reported breast cup size, ranging from A to DDD/F. The majority of participants fell into the B (n = 81), C (n = 62), and D (n = 33) cup size categories. The distribution of anthropometric and clinical variables by cup size is presented in Table 1.

The age distribution of the study cohort: The largest age group was women aged 21–25 years, comprising 67 participants (33.5% of the total cohort). This was followed by the 31–36-year-old group, with 56 participants (28%), and the 26–30-year-old group, with 40 participants (20%). Women aged 18–20 accounted for 18.5% of the cohort (37 participants). These findings demonstrate that the study population predominantly consisted of young adult women aged 21 to 36 years, representing the primary demographic for evaluating the relationship between breast size and back pain in this cohort.

Women with larger cup sizes tended to be older and heavier, with a notable increase in bra band length. The mean age rose from 24.0 ± 5.0 years in the B cup group to 28.1 ± 6.0 years in the DD/E group. Weight similarly increased from 59.6 ± 9.8 kg to 84.9 ± 12.0 kg across the same categories. Bra width rose progressively, averaging 74.8 ± 4.8 cm in the B group and 85.8 ± 4.4 cm in the DD/E group. Height remained relatively stable across the groups (approximately 1.6–1.7 m).

Pain distribution varied substantially by cup size. Only 3.7% of women in the B cup group reported lumbar pain, compared to 65% in the DD/E group. Thoracic pain was reported by 1.2% in the B group and 70% in the DD/E group, while cervical pain rose from 2.5% to 80% across the same categories. Total pain-free status declined markedly with breast size: 95.1% of the B cup participants reported no backache, compared to just 15% in the DD/E group. Pain severity, as measured by the visual analog scale (VAS), increased in parallel with cup size, from a mean of 0.3 ± 1.6 in the B cup group to 6.0 ± 2.9 in the DD/E group. The VAS score in cup A is significantly biased by the small cohort size, with one participant reporting a VAS score of 9. The total number of days with pain per year similarly increased from 4.6 ± 40.5 to 38.3 ± 111.7.

Analysis of the SF-12 quality of life domains revealed a significant association between breast size and specific functional outcomes among participants reporting back pain. Specifically, a chi-square test demonstrated a significant relationship between cup size and work-related limitations (*p* = 0.006), with women in the larger cup size groups reporting greater interference with occupational functioning. Similarly, social functioning was significantly affected by breast size (*p* < 0.001), with higher social disturbance scores observed among women with larger breasts. In contrast, no significant associations were identified between cup size and self-reported calmness *(p* = 0.973), energy levels (*p* = 0.260), or attrition scores (*p* = 0.103), indicating that while breast size influenced physical and social aspects of quality of life, its impact on emotional well-being and fatigue was less pronounced. The average social disturbance score did not change considerably with cup size, increasing from 4.9 ± 0.5 in the B group to 5.0 in the DDD/F group.

A binary logistic regression model assessing the relationship between bra band size and the presence of any backache (lumbar, thoracic, or cervical) revealed a statistically significant association. Each 1 cm increase in band length conferred a 19.8% increase in the odds of experiencing back pain (OR = 1.198, *p* < 0.001). The findings remained significant when this model was applied separately to individual spinal regions. Lumbar, thoracic, and cervical pain odds increased by 15.4%, 18.9%, and 20.9% for each centimeter increase in bra width (all *p* < 0.001).

Cup size was also significantly associated with back pain in a regression model (Wald χ^2^ = 51.369, *p* < 0.001) (Table 2). Although not all categories reached individual significance, a clear dose-response trend was observed. The participants with the largest cup size exhibited a 12-fold increase in the odds of experiencing back pain compared to the reference group (OR = 12.0, *p* = 0.071), while even a modest increase to B cup size yielded a 1.8-fold increase in the odds (Table 2).

Weight and age were significant predictors of back pain in univariate analyses. Each kilogram of weight increased the odds by 8.2% (OR = 1.082, *p* < 0.001), and each year of age increased the odds by 11.6% (OR = 1.116, *p* < 0.001). Height was not significantly associated with back pain (*p* = 0.930).

A multivariate logistic regression incorporating age, weight, and cup size demonstrated that the model was significant (Wald χ^2^ = 33.350, *p* < 0.001). However, in this model, age (*p* = 0.218) and weight (*p* = 0.392) lost significance, while the contribution of cup size remained robust. These findings suggest the presence of collinearity, likely due to the strong correlation between breast size and body weight.

A subgroup analysis of the 79 participants reporting back pain was conducted to explore quality of life outcomes. A chi-square test revealed a significant association between cup size and work limitations (*p* = 0.006). In contrast, no significant associations were found between cup size and calmness (*p* = 0.973), energy (*p* = 0.260), or attrition (*p* = 0.103). However, a highly significant association was found between cup size and social disturbance score (*p* < 0.001), suggesting that breast size influences perceived social functioning more than psychological or fatigue-related domains.

No relationship was observed between menstrual pain and cup size (*p* = 0.828). Excluding women with menstrual pain from the multivariate model did not meaningfully change the results. Cup size remained a significant predictor (Wald χ^2^ = 26.311, *p* < 0.001), while age and weight failed to reach statistical significance again.

## 4. Discussion

This study investigated the association between female breast size and backache complaints, as well as related functional and quality of life outcomes in a young adult female population. The results demonstrate a significant and consistent association between larger breast size, measured by cup size and bra band length, and increased prevalence, duration, and severity of back pain. These associations were significant in the thoracic and cervical regions, accompanied by greater functional limitations and impaired social participation.

Among participants with a B cup, 95.1% reported no backache, while only 15% of those with DD/E cups were pain-free. Thoracic and cervical pain prevalence increased from 1.2% to 70% and 2.5% to 80%, respectively, across the same cup size range. VAS pain scores also increased markedly, from 0.3 ± 1.6 in the B group to 6.0 ± 2.9 in the DD/E group, with a mean pain duration rising from 4.6 to 38.3 days per year. These figures illustrate a robust, dose-dependent relationship between breast size and symptom burden.

Binary logistic regression confirmed these trends. Each 1 cm increase in bra band length was associated with a 19.8% increase in the odds of back pain (OR = 1.198, *p* < 0.001). When analyzing spinal regions separately, bra width remained a strong predictor of lumbar (OR = 1.154), thoracic (OR = 1.189), and cervical pain (OR = 1.209), all statistically significant. Cup size also emerged as a key categorical predictor of back pain. In the multivariate model, participants with the largest cup size exhibited a twelvefold increase in the odds of backache compared to the reference group (OR = 12.0, *p* = 0.071), and even a shift to a B cup doubled the odds (OR = 1.8).

These findings are consistent with prior studies demonstrating that breast hypertrophy contributes to increased musculoskeletal strain. Coltman et al. reported that women with breast volume > 1200 mL experienced significantly higher musculoskeletal pain scores across the upper torso [3], while McGhee et al. observed greater thoracic kyphosis and reduced shoulder and scapular function in women with larger breasts [4]. Similarly, Schinkel-Ivy and Drake demonstrated that increased breast size leads to altered spinal kinematics and postural muscle activation [7], providing a plausible biomechanical mechanism.

In univariate analysis, weight and age also emerged as significant predictors of back pain, with each kilogram of weight and one year of age associated with 8.2% and 11.6% increases in odds, respectively. However, in the multivariate model that included cup size, these associations were no longer significant, likely due to collinearity and confounding, as weight and cup size are positively correlated. This reinforces the interpretation that breast size, rather than general body mass, may be the primary biomechanical driver of pain in this population.

The functional impact of breast size was clearly reflected in the SF-12 quality of life domains in this study. Women with larger breasts reported significantly greater limitations in their ability to perform work-related tasks (*p* = 0.006), and their social functioning scores were also significantly impaired (*p* < 0.001). These findings align with existing literature emphasizing that breast hypertrophy imposes limitations that extend beyond physical pain to tangible disruptions in daily functioning and social engagement. For example, Mian et al. demonstrated that reduction mammaplasty leads to significant improvements in physical functioning, social participation, and overall quality of life [14]. Meanwhile, Goulart et al. reported that surgical reduction in breast size not only alleviates pain but also improves posture and decreases the perception of discomfort [16].

Interestingly, the present study found no significant association between breast size and psychological or emotional well-being domains, including calmness (*p* = 0.973), energy levels (*p* = 0.260), and mental strain as measured by attrition (*p* = 0.103). This suggests that the burden imposed by larger breasts in this young, otherwise healthy population is primarily mechanical and social, rather than psychological. These results align with previous work by Spencer et al., who found that while larger breast size was associated with musculoskeletal discomfort, it did not directly influence psychological well-being [11]. Additionally, no association was observed between breast size and menstrual pain (*p* = 0.828), indicating that cyclical pain does not confound the relationship between breast size and back pain in this cohort. This interpretation is further supported by the finding that exclusion of women with menstrual complaints from multivariate models did not alter the strong, statistically significant association between breast size and backache (*p* < 0.001).

Collectively, these findings emphasize that large breast size adversely affects physical functioning and social participation, with minimal psychological or emotional impact detected in this population. The data support the argument that reduction mammaplasty, beyond cosmetic considerations, may play a meaningful role in improving physical and social aspects of quality of life in women with breast hypertrophy.

This study has several limitations. First, breast size was estimated based on self-reported bra cup and band size, which may not accurately reflect breast volume or fit. Misreporting or variability in sizing standards across brands could introduce measurement errors. Objective anthropometric or imaging-based assessments would provide a more precise evaluation. Second, the study’s cross-sectional nature limits causal inference; although breast size is strongly associated with pain, the temporality of this association cannot be established. Longitudinal follow-up would help confirm whether increasing breast size precedes the onset of back pain. Third, the study population was limited to women attending orthopedic clinics and may not represent the general female population. Additionally, specific confounders, such as physical activity level, occupational demands, and spinal anatomy, were not controlled for and may have influenced the outcomes.

## 5. Conclusions

In conclusion, the results of this study strongly support the hypothesis that larger breast size is significantly associated with increased backache and reduced quality of life in young women. Both breast cup size and band length were predictive of lumbar, thoracic, and cervical pain, with up to twelvefold increased odds observed in women with the largest breasts. These findings demonstrate that breast size plays a significant, independent role in musculoskeletal symptomatology. Clinicians should consider breast size when evaluating back pain in young women, and quality of life impacts, particularly social and occupational limitations, should be recognized as part of the clinical burden. Future studies should aim for a prospective design and incorporate objective breast measurements to validate and expand upon these findings. Surgical intervention, such as reduction mammaplasty, may be justified not only for cosmetic concerns but as a legitimate medical treatment for backache and functional impairment.

## Figures and Tables

**Table 1:** Cohort characteristics of 200 participants.

Cup Size	A	B	C	D	DD/E	DDD/F	*p*-Value
Cohort Size (no)	3	81	62	33	20	1	
Age (y)	24.3 ± 4	24 ± 5	26.7 ± 4.9	28.4 ± 5.6	28.1 ± 6	32	<0.001
Weight (kg)	60.3 ± 4.2	59.6 ± 9.8	67.3 ± 9.8	77.1 ± 11.4	84.9 ± 12	90	<0.001
Height (m)	1.6 ± 0.1	1.6 ± 0.1	1.7 ± 0	1.7 ± 0.1	1.7 ± 0.1	1.59	0.202
Band Width (cm)	71.7 ± 2.9	74.8 ± 4.8	80.4 ± 5.6	81.4 ± 3.6	85.8 ± 4.4	85	<0.001
Pain Free no(%)	2 (66.7)	77 (95.1)	32 (51.6)	7 (21.2)	3 (15)	0 (0)	<0.001
Lumbar Pain no(%)	1 (33.3)	3 (3.7)	18 (29)	12 (36.4)	13 (65)	0 (0)	<0.001
Thoracic Pain no(%)	1 (33.3)	1 (1.2)	20 (32.3)	15 (45.5)	14 (70)	1 (100)	<0.001
Cervical Pain no(%)	0 (0)	2 (2.5)	21 (33.9)	21 (63.6)	16 (80)	1 (100)	<0.001
Menstrual Pain no(%)	0 (0)	2 (2.5)	11 (17.7)	8 (24.2)	5 (25)	0 (0)	0.004
Total Days of Pain	0.3 ± 0.6	4.6 ± 40.5	24.5 ± 90.2	34.8 ± 106.1	38.3 ± 111.7	1	<0.001
VAS Score	3 ± 5.2	0.3 ± 1.6	3.2 ± 3.3	5.8 ± 2.9	6 ± 2.9	8	<0.001
Health Perception	2 ± 1.7	1.1 ± 0.4	1.7 ± 0.9	2.2 ± 0.8	2.2 ± 0.9	2	<0.001
Affects Work	0.3 ± 0.6	0 ± 0.2	0.1 ± 0.2	0.2 ± 0.4	0.5 ± 0.5	0	<0.001
Calmness	2 ± 1.7	1.1 ± 0.6	2.2 ± 1.4	2.8 ± 1.4	3 ± 1.4	2	<0.001
Energy Score	2 ± 1.7	1.2 ± 0.8	2.1 ± 1.4	2.5 ± 1.2	3.2 ± 1.3	2	<0.001
Attrition	4.7 ± 2.3	5.9 ± 0.6	5.1 ± 1.3	5 ± 1	4.4 ± 1	5	<0.001
Social Disturbance Score	4 ± 1.7	4.9 ± 0.5	4.7 ± 0.8	4.8 ± 0.5	4.3 ± 1	5	<0.001

**Table 2:** Odds ratios (ORs) with 95% confidence intervals (CIs) and *p*-values.

Variable	OR	95% CI	*p*-Value
Bra band width (per 1 cm increase)	1.198	1.126–1.275	<0.001
Cup size-B vs. Reference	1.8	0.9–3.6	0.088
Cup size-C vs. Reference	7.429	0.7–78.2	0.122
Cup size-D vs. Reference	12	0.9–162.4	0.071
Cup size-DD/E vs. Reference	12	0.9–162.4	0.071
Weight (per 1 kg increase)	1.082	1.051–1.115	<0.001
Height (per 1 m increase)	1.264	0.01–138.0	0.93
Age (per 1 year increase)	1.116	1.056–1.179	<0.001

## Data Availability

The datasets generated during and/or analyzed during the current study are available from the corresponding author upon reasonable request.
